# Rapid Detection of Antibiotic Mycelial Dregs Adulteration in Single-Cell Protein Feed by HS-GC-IMS and Chemometrics

**DOI:** 10.3390/foods14101710

**Published:** 2025-05-12

**Authors:** Yuchao Feng, Yang Li, Wenxin Zheng, Decheng Suo, Ping Gong, Xiaolu Liu, Xia Fan

**Affiliations:** 1 Institute of Quality Standards and Testing Technology for Agro-Products of Chinese Academy of Agricultural Sciences, Beijing 100081, China; fengyuchao0321@126.com (Y.F.); liyang@caas.cn (Y.L.); suodecheng@caas.cn (D.S.); liuxiaolu@caas.cn (X.L.); 2 Institute of Animal Husbandry Quality Standards, Xinjiang Academy of Animal Science, Urumqi 830057, China; zwx2020@126.com (W.Z.); ggpp99@foxmail.com (P.G.)

**Keywords:** volatile compounds, headspace-gas chromatography-ion mobility spectrometry, chemometric method, antibiotic mycelial dregs, protein feed

## Abstract

Single-cell protein feed (SCPF) is an important supplement to protein feed materials, but its authenticity is often affected by antibiotic mycelial dregs (AMD). Headspace-gas chromatography–ion mobility spectrometry (HS-GC-IMS), integrated with chemometrics, was utilized to differentiate nucleotide residue (NR), three AMDs, and adulterated samples with concentrations ranging from 0.1% to 20% (*w*/*w*). Orthogonal partial least squares discriminant analysis (OPLS-DA) and principal component analysis (PCA) were applied to classify the adulterated samples. In addition, the feasibility of quantitative analysis of the AMDs content in adulterated SCPF based on partial least squares regression (PLSR) algorithm. In total, 88 volatile organic compounds (VOCs) were detected. The differences in VOCs between NR and AMD mainly came from aldehydes, alcohols, and esters. The OPLS-DA models effectively identified AMD in adulterated NR samples (Accuracy = 100%), demonstrating the HS-GC-IMS data’s good application potential for the SCPF adulteration. Nine VOCs, i.e., 2-ethyl-3-methylpyrazine, dihydro-5-methyl-2(3H)-furanone, 2-methylpropanol, (E,E)-2,4-heptadienal, linalool, 2,3,5-trimethylpyrazine, citronellol, acetoin, and 3-methylbutan-1-ol, were proposed as key markers for detecting NR adulterated with AMDs. The PLSR algorithm was further used to determine the AMD content in NR (*R*^2^_cal_ = 0.96, *R*^2^_cv_ = 0.94). This study validated HS-GC-IMS’s ability to analyze volatile organic compounds in feed and showcased its utility as a convenient, quick, and affordable tool for SCPF authenticity screening.

## 1. Introduction

In recent years, there has been a growing demand for the extensive implementation of soybean meal reduction and replacement operations in China. In grain and feed safety, replacing soybean meal with unconventional protein feed has become a significant industry trend. Single-cell protein feed (SCPF) has the advantages of abundant raw materials, short production cycles, high production efficiency, production without seasonal climate constraints, and concentrated fermentation [1]. SCPF is often studied as an alternative protein feed, but its development and utilization have numerous risks [2]. Feed and livestock product safety issues caused by the use of prohibited additives have become increasingly prominent, which will not only threaten the health of people and livestock but also greatly reduce the nutritional and economic value of feed products.

Antibiotic mycelial dregs (AMD) are an inexpensive, protein-rich industrial waste byproduct of antibiotics [3]. The content of the conventional chemical components (e.g., crude protein, crude fat, and crude ash) of AMD is similar to that of SCPF, but the presence of residual antibiotics and unidentified metabolic products from AMD has introduced substantial risks to the breeding industry [4]. Fueled by economic interests with the shortage of protein feed materials and their continuous rise in market price, some companies illegally add AMD residue to SCPF or use it as a substitute. For this reason, there is an urgent need to develop a rapid detection method suitable for the identification of SCPF authenticity and adulteration.

The similarities between AMD and protein feed material make them difficult to distinguish. Current approaches primarily target the detection of trace antibiotics or antibiotic residues, employing analytical techniques such as high-performance liquid chromatography (HPLC) [5], HPLC coupled with mass spectrometry [6], gas chromatography–mass spectrometry (GC-MS) [7], and enzyme-linked immunosorbent assay [8]. However, these methods heavily rely on expensive instruments with high analysis costs and typically result in the production of environmentally unfriendly compounds. Meanwhile, microscopic near-infrared/infrared imaging has been used to detect the fraudulent addition of AMD to cottonseed meal, soybean meal, and distillers’ dried grains with solubles [9], while hyperspectral imaging has been employed to check the authenticity of feed protein materials [10]. These methods, combined with chemometrics, could detect as little as 1% adulteration (*w*/*w*). However, these methods collect huge amounts of data and use complex statistical procedures to construct adulteration models. Furthermore, the reliance on costly equipment and trained experts may impede their implementation in quality inspection agencies [11].

Each protein feed material has a distinct odor comprising hundreds of volatile organic compounds (VOCs). Thus, changes in odor are one of the most sensitive indicators of protein feed material quality [12]. VOCs from protein feed materials can indicate sample data, showing the link between VOC changes and feed quality [13]. For agri-food volatile analysis, GC-MS is the gold standard, identifying chemicals by comparing retention times to standards. However, its time-consuming nature and complex sample prep limit its use for rapid or on-site screening [14].

Headspace-gas chromatography-ion mobility spectroscopy (HS-GC-IMS) is a state-of-the-art method for characterizing and measuring volatile compounds in diverse matrices [15]. It separates gas-phase ions based on their drift tube mobility under a constant electric field at atmospheric pressure [16], enabling 2D separation of volatiles through the combination of GC retention time and IMS drift time [15]. Due to its ease of sample preparation, high sensitivity, resolution, operational efficiency, and flavor visualization, HS-GC-IMS has been widely adopted for authenticity analysis in agricultural products, such as milk [17], honey [18], oil [19], juices [20], flour [21], and traditional Chinese medicine [22]. This study is the first to employ HS-GC-IMS for detecting AMD adulteration in SCPF and quantifying adulterant levels.

This study focused on developing an accurate and sensitive approach to rapidly detect AMD adulteration in SCPF. First, HS-GC-IMS was employed to analyze VOCs, uncovering sample differences through volatile fingerprints. Subsequently, orthogonal partial least squares discriminant analysis (OPLS-DA) and principal component analysis (PCA) were utilized to classify adulterated samples, while partial least squares regression (PLSR) was applied to predict AMD concentrations in SCPF. The study aims to elucidate flavor changes in SCPF following AMD adulteration and provide a reference for the rapid identification of AMD-adulterated protein feed materials.

## 2. Materials and Methods

### 2.1. Sample Collection and Preparation

Ten nucleotide residues (NR01–NR10) were obtained from feed-producing companies in the Shandong and Henan provinces. Five oxytetracycline dregs (OD01–OD05), five streptomyces dregs (SD01–SD05), and five avermectin dregs (AD01–AD05) were collected from Hebei and Shandong provinces. Nucleotide residue (NR) is the most commonly used single-cell protein feed on the market, while oxytetracycline dregs (OD), streptomyces dregs (SD), and avermectin dregs (AD) have relatively large yields of antibiotic residues. However, during the manufacturing process, their raw materials and physical properties are similar. The samples were preserved at −20 °C until use.

OD adulterants were formulated by blending OD and NR at ratios of 0.1:99.9, 0.5:99.5, 1:99, 5:95, 10:90, and 20:80 (*w*/*w*), and SD or AD adulterants were created by mixing SD or AD with NR at the same ratios. Four samples were prepared at each concentration for a total of 72 adulterated samples in three categories.

### 2.2. HS-GC-IMS Measurement

The sample volatiles were analyzed using an IMS instrument (FlavourSpec, Gesellschaft für analytische Sensor systeme mbH, Dortmund, Germany) with an internally installed Agilent 990 gas chromatograph (Agilent Technologies, Palo Alto, CA, USA), referring to the methods of previous reports with some modifications [23].

Samples (2.0 g) were weighed, placed in a 20-mL headspace glass vial, and incubated at 80 °C for 20 min. Subsequently, 500 μL of headspace gas was injected into the injector (85 °C, splitless mode) and driven into an FS-SE-54-CB-1 capillary column (15 m × 0.53 mm, Agilent Technologies) under isothermal conditions at 60 °C, using high-purity nitrogen (99.999%) as the carrier gas. The carrier gas was passed through the HS-GC-IMS injector, and the sample was transferred to the GC column as follows: 2 mL/min for 2 min, 10 mL/min for 8 min, 100 mL/min for 10 min, and 150 mL/min for 10 min until the flow stopped. After GC separation, the analytes were ionized in the IMS ionization chamber in the positive ion mode by a β-radiation-3H ionization source with 300 MBq activity. The ions were driven into the drift tube at 45 °Con a constant tube linear voltage (400 V/cm) with a nitrogen flow of 150 mL/min as the drift gas. The drifted ions were captured in 30 ms. A total of 97 HS-GC-IMS data (biological replication) were obtained.

One milliliter of a mixture of *n*-ketones C4–C9 (10 mg/L, Sinopharm Chemical Reagent Co., Ltd., Beijing, China) was added to a headspace glass vial and analyzed under identical conditions as the samples. A calibration curve was established based on the retention index and retention time of the *n*-ketones, which was then used to calculate the retention index of volatile compounds. IMS data were processed using Laboratory Analytical Viewer software, GC-IMS Library Search, and the Reporter and Gallery plot plug-ins.

### 2.3. Data Processing

PCA is a dimensionality reduction technique that maps information from the original variables onto a smaller number of latent variables, termed principal components [24]. It produces a visual depiction of sample-variable relationships, revealing how measured variables influence similarities or differences between samples. Hence, PCA (using 94 signal peaks) was used to visualize the spectral differences between NR and antibiotic mycelial dregs in adulterated samples to observe whether specific clustering trends existed in each group.

OPLS-DA was developed as a refined version of PLS-DA to distinguish between two or more groups (classes) using multivariate data [25]. In contrast to PLS-DA, OPLS-DA relies on one component for class prediction, with the other components describing variation orthogonal to the first predictive component [26]. The model was evaluated by the goodness of fit (*R*^2^ (cum)), predictive ability (cross-validated *Q*^2^ (cum)), and correct classification rate of the samples [25]. *R*^2^ (cum) and *Q*^2^ (cum) were, respectively, the cumulative percentage of the variation of the dependent variable explained by the model and a measure of the predictive ability [27]. Both values range between 0 and 1, with a higher value indicating a better fit of the model, and with a significance threshold of 0.5 being considered generally acceptable. To assess the importance of VOCs in explaining the classification model, a Variable Importance in the Projection (VIP) was created [28]. The VIP value for each variable was determined by weighting the squared OPLS loading against the explained sum of squares for each model component.

The PLSR model (using 94 signal peaks) was established through a linear regression model between the variable matrices Y and X. The predicted results were achieved by extracting a set of orthogonal factors with powerful predictive ability [29]. The coefficient of determination for calibration (*R*^2^), root mean square error of estimation (RMSEC), and root mean square error of verification (RMSECV) parameters were calculated to assess the model performance.

Data analysis was performed using MATLAB R2015a (MathWorks, Inc., Natick, MA, USA) with PLS_Toolbox 7.1 (Eigenvector Research, Wenatchee, WA, USA) for PCA and PLSR models, and SIMCA-P 14.1 (Umetrics, Umeå, Sweden) for OPLS-DA analysis.

## 3. Results and Discussion

### 3.1. Topographic Plots

**Figure 1:**
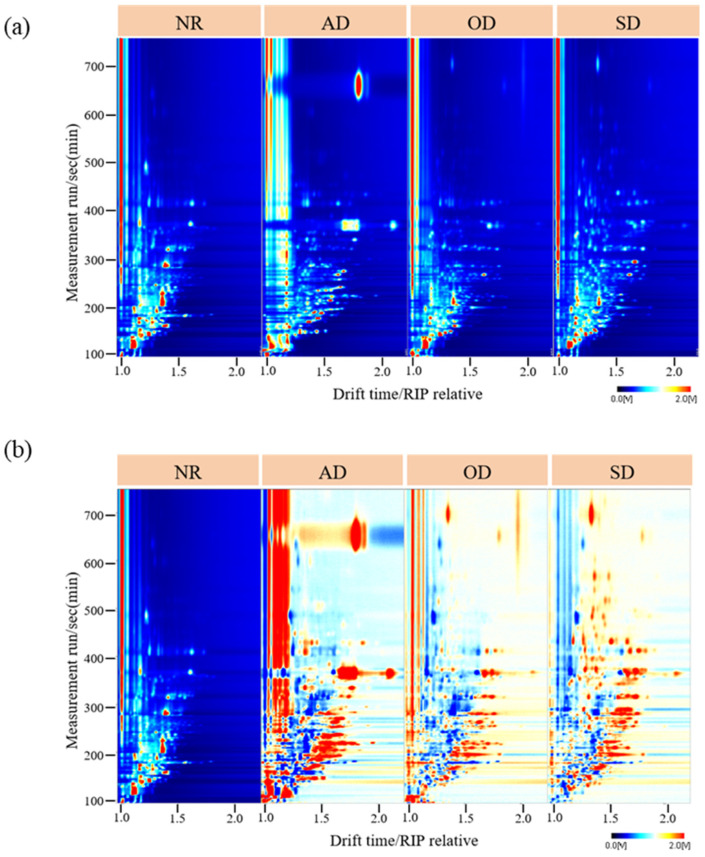
(**a**) Two-dimensional (2D) topographic plots of NR and AMD; (**b**) Subtraction topographic plots between NR and AMD.

Figure 1a shows the topographic plots of the NR and AMD samples from HS-GC-IMS results. The overall GC-IMS topographic plot was blue, with the vertical coordinate representing the GC retention time (s) and the horizontal coordinate representing the IMS drift time. The red vertical line at abscissa 1.0 represented the reactive ion peak after normalization. Each point in the topographic plot indicated a volatile compound, and its color represented the content of volatile compounds from low (white) to high (red). From the topographic plots, we observed the total VOCs of NR, AD, OD, and SD, as well as the differences in VOCs between samples. Most signals appeared within the drift time of 1.00–2.00 ms and the retention time of 100–700 s. As shown in the VOC plots, the VOC number of the AMD samples, especially AD, was significantly higher than that of the NR samples. Topographic plot variations among pure samples indicated potential differences in their volatile profiles.

To observe these differences more clearly, the AMD samples were compared using the NR sample as a reference to obtain the differential spectra (Figure 1b). If the topographic map background, after deduction using reference samples, appeared white, it indicated that the VOC content was identical. Red and blue colors indicated that the concentration of the tested sample was higher (Red) or lower (Blue) than the reference. AD provided the most red dots, followed by SD and OD, indicating that the difference between AD and NR was the largest. The 2D topographic plot reveals HS-GC-IMS’s potential to distinguish NR from AMD using their volatile compounds.

### 3.2. Fingerprint Analysis of NR and AMD

**Figure 2:**
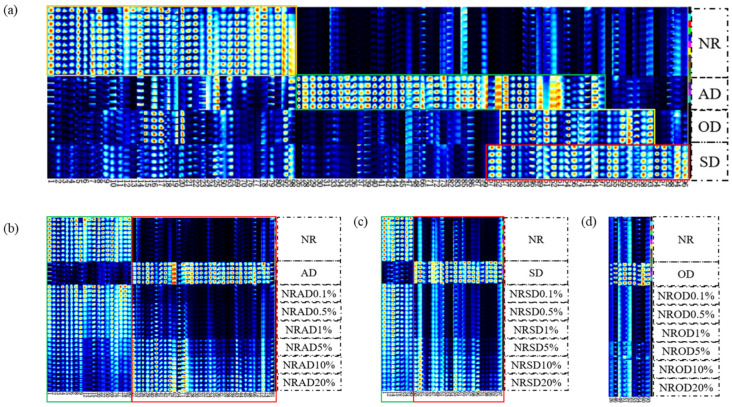
Fingerprint plots of pure and adulterated samples; (**a**) NR and AMD; (**b**) AD-adulterated, (**c**) SD-adulterated, and (**d**) OD-adulterated samples.

To differentiate between substances in NR and AMD, fingerprints were manually generated for all peaks to be analyzed in the obtained 2D topographic plots (Figure 2a). Rows indicate signal peaks for individual samples, and columns reflect the peaks of identical VOCs in multiple samples. Some compounds may exhibit dual or multiple signals due to concentration differences. In total, 88 volatile organic compounds (94 signal peaks) were detected, including 19 aldehydes, 16 alcohols, 18 esters, 13 ketones, seven acids, six heterocyclic compounds, and nine others, as shown in Table 1.

### 3.3. Discriminant Analysis of Adulterated Samples

There were 36 VOCs with high content in NR, namely seven aldehydes, five alcohols, four esters, six ketones, four acids, and 10 others (Figure 2a, orange box). A total of 45 VOCs in AD (eight aldehydes, eight alcohols, 12 esters, six ketones, two acids, and nine others, Figure 2a, green box), 22 VOCs in OD (six aldehydes, five alcohols, three esters, three ketones, two acids, and three others, Figure 2a, yellow box), and 29 VOCs in SD (six aldehydes, six alcohols, six esters, four ketones, two acids, and six others, Figure 2a, red box) had significantly higher content than in NR. These differences in VOC content between NR and AMD mainly came from aldehydes, alcohols, and esters.

To directly reflect the dynamic changes of VOCs in adulterated samples, the volatile fingerprints were constructed (Figure 2b–d). The VOC content did not always change with the increase in AMD concentration. For AD-adulterated samples, the content of 19 VOCs decreased with the increase in AD concentration (Figure 2b, green box), while 32 VOCs gradually increased in content (Figure 2b, red box). A 0.1% AD-adulterated sample could be distinguished from NR by alpha-phellandrene (27). For SD-adulterated samples, the content of seven VOCs decreased with the increase in SD concentration (Figure 2b, green box), while 20 VOCs gradually increased in content (Figure 2c, red box). A 0.1% SD-adulterated sample could be distinguished from NR by benzaldehyde (60). In OD-adulterated samples, only nine VOCs increased in content with increasing OD concentration, and a 5% OD-adulterated sample could be distinguished from NR. Thus, HS-GC-IMS had a better identification ability for AD- and SD-adulterated samples compared with OD-adulterated samples

#### 3.3.1. Exploratory Analysis Using PCA

**Figure 3:**
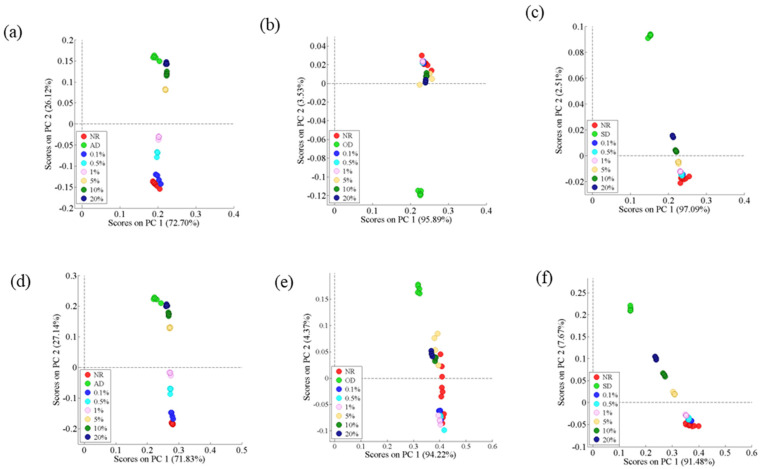
PCA scatter plots. All signal peaks: (**a**) NR and AD-adulterated samples; (**b**) NR and OD-adulterated samples; (**c**) NR and SD-adulterated samples. Characteristic signal peaks: (**d**) NR and AD-adulterated samples; (**e**) NR and SD-adulterated samples; (**f**) NR and OD-adulterated samples.

The PCA score plots for the classification using standardized HS-GC-IMS data are presented in Figure 3a–c, with the first two principal components explaining 98.82%, 99.42%, and 99.60% of the AD, OD, and SD spectral data, respectively. The best separation was observed between NR and AMD in either the first or fourth quadrants. As shown in Figure 3a, samples with a low percentage of adulteration were plotted closer to NR, whereas those with a high percentage of adulteration were plotted closer to AD. However, PCA was not effective in distinguishing NR from samples with a low percentage (0.1–1%) of adulteration (Figure 3b,c).

The score plots of PCA using 51 (Figure 2b), 27 (Figure 2c), and nine (Figure 2d) signal peaks (standardized) are, respectively, shown in Figure 3d–f. The first two principal components accounted for 98.97%, 98.59%, and 99.15% of AD, OD, and SD spectral data, respectively. PCA still clearly distinguished the NR and AD-adulterated samples, and the characteristic signal peaks improved the ability of PCA to distinguish the NR and SD-adulterated samples (1–20%). In contrast, there was no clear separation between the NR and OD-adulterated samples.

#### 3.3.2. Exploratory Analysis Using OPLS-DA

**Figure 4:**
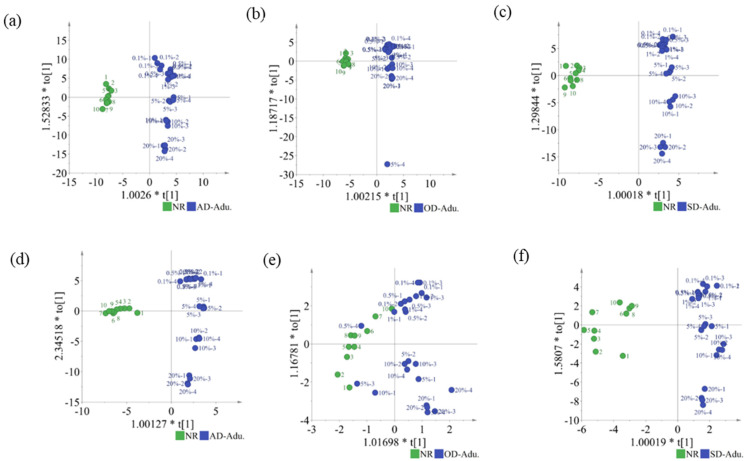
OPLS-DA scatter plots. All signal peaks: (**a**) NR and AD-adulterated samples; (**b**) NR and OD-adulterated samples; (**c**) NR and SD-adulterated samples. Characteristic signal peaks: (**d**) NR and AD-adulterated samples; (**e**) NR and OD-adulterated samples; (**f**) NR and SD-adulterated samples.

OPLS-DA sharpens the analysis of discriminant variation by filtering out data variation unrelated to class separation. The score plots of the thrice cross-validated OPLS-DA models, built using the HS-GC-IMS datasets (94 signal peaks) of NR and the adulterated samples, are shown in Figure 4a–c. The *R*^2^(cum) and *Q*^2^(cum) values of the OPLS-DA models are shown in Table 2. For the OPLS-DA models for AD, SD, and OD, *R*^2^*X*(cum) = 0.851, 0.692, and 0.974, *R*^2^*Y*(cum) = 0.976, 0.987, and 0.933, and *Q*^2^(cum) = 0.963, 0.971, and 0.908, respectively. The cumulative *R*^2^*X*(cum), *R*^2^*Y*(cum), and *Q*^2^(cum) values indicated the good predictive ability of the OPLS-DA models. The NR samples were clustered to the left of the central axis, whereas the NR samples adulterated with AMD were grouped on the right. The correct classification rate of samples from the dataset was 100% for NR and adulterated samples. Using microscopic infrared imaging, it is possible to identify 1% adulterated samples, which is higher than the lowest adulteration concentration (0.1%) that can be identified based on HS-GC-IMS. These results demonstrated that the HS-GC-IMS is suitable for the authenticity analysis of SCPF without the need for sample preparation, which makes this approach fast and cost-effective. As shown in Figure S1, the *y*-intercepts of *Q^2^* were less than 0, indicating that the classification models were not over-fitted.

Variables with a VIP value greater than 1 were considered to play a crucial role in the discrimination method. Thus, VOCs with VIP > 1 for the three OPLS-DA models were screened (Figure S2). In the AD-Adu. model, a total of 44 VOCs significantly contributing to the model (VIP > 1), namely 6 alcohols, 8 aldehydes, 10 ketones, seven esters, five acids, three heterocyclics, and five others, were identified. Among them, citronellol had the highest contribution, while 2-methylpropanol, 2,3,5-trimethylpyrazine, linalool, and dihydro-5-methyl-2(3H)-furanone were ranked very highly. In the SD-Adu. model, 45 VOCs with VIP > 1, namely 9 alcohols, 8 aldehydes, six ketones, six esters, five acids, three heterocyclics, and seven others, were identified. Notably, bihydro-5-methyl-2(3H)-furanone, 3-methylbutan-1-ol, linalool, (*E*,*E*)-2,4-heptadienal, and acetoin had the highest VIP values. For the OD-Adu. model, 35 VOCs with VIP > 1, namely 7 alcohols, 8 aldehydes, four ketones, five esters, three acids, two heterocyclics, and eight others, were identified. 2-Ethyl-3-methylpyrazine had the highest VIP value, with acetoin, dihydro-5-methyl-2(3H)-furanone, citronellol, and 3-methylbutan-1-ol ranking very highly. These volatile compounds are thought to be vital for distinguishing NR from AMD-adulterated materials.

OPLS-DA models, built using 51 (Figure 2b) and 27 (Figure 2c) signal peaks, were, respectively, used to discriminate NR and AD-/SD-adulterated samples (Figure 4d,f). However, the OPLS-DA model using nine signal peaks (Figure 2d) showed a lower correct classification rate (95% accuracy) for OD-adulterated samples (Figure 4e).

### 3.4. Quantitative Analysis of Adulterated Samples

**Figure 5:**
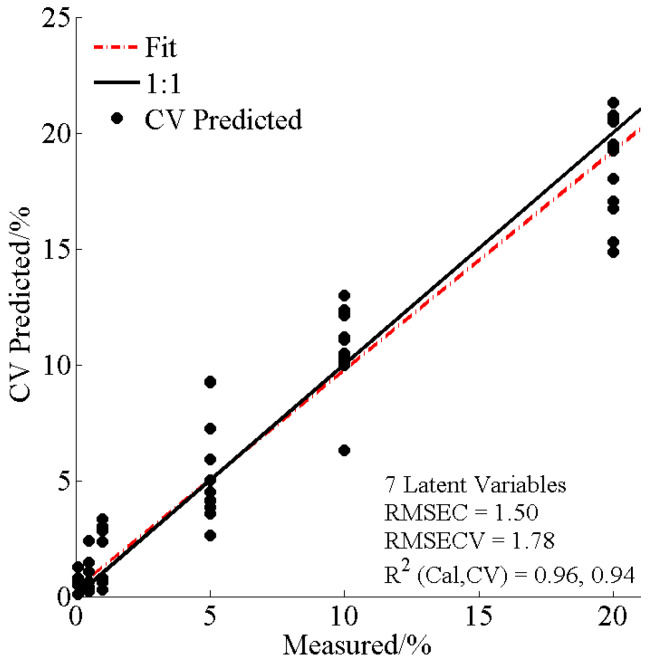
Predicted levels of AMD in NR by PLSR.

After confirming AMD presence in adulterated SCPF via the OPLS-DA model, a PLSR-based model was created to quantify AMD levels in the samples. The PLSR model was obtained with seven latent variables for 72 adulterated samples (0.1–20%) (Figure 5). According to the *R*^2^_cal_ (0.96), *R*^2^_cv_ (0.94), RMSEC (1.50), and RMSECV (1.78) values, the quantitative model for AMD in SCPF was considered suitable.

## 4. Conclusions

This study examined the performance of HS-GC-IMS in the rapid identification of antibiotic mycelial dregs in single-cell protein feed. Among the 94 signals detected, 88 single compounds were identified and classified into seven groups: aldehydes, alcohols, esters, ketones, acids, heterocyclics, and others. Differences in the volatile organic compound content of nucleotide residue and antibiotic mycelial dregs mainly came from aldehydes, alcohols, and esters. The orthogonal partial least squares discriminant analysis models built using the HS-GC-IMS data of nucleotide residue and adulterated samples achieved a high classification rate even when the adulteration concentration was 0.1%. Nine volatile organic compounds, i.e., 2-ethyl-3-methylpyrazine, dihydro-5-methyl-2(3H)-furanone, 2-methylpropanol, (*E*,*E*)-2,4-heptadienal, linalool, 2,3,5-trimethylpyrazine, citronellol, acetoin, and 3-methylbutan-1-ol, were proposed as key markers for detecting nucleotide residue adulterated with antibiotic mycelial dregs. The analysis demonstrated that HS-GC-IMS, coupled with chemometrics, offers a swift, reliable, and straightforward method for assessing the adulteration and authenticity of single-cell protein feed. Given its cost-effectiveness and portability, HS-GC-IMS could emerge as a valuable tool for identifying adulteration in protein feed materials.

HS-GC-IMS is a promising technique for distinguishing antibiotic mycelial dregs from single-cell protein feed, but its effectiveness may be influenced by feed sample characteristics like type, size, and representativeness. Additionally, VOC profiles can differ under varying environments or storage conditions. Future studies should address these issues to improve the method’s reliability and robustness.

## Figures and Tables

**Table 1:** Volatile compound data obtained using HS-GC-IMS.

Number	Compound	CAS#	Formula	MW ^a^	RI ^b^	Rt ^c^	Dt ^d^
Aldehydes							
3	3-(methylthio)propanal (methional)	3268-49-3	C_4_H_8_OS	104.2	909.6	281.917	1.39214
7	Trans-2-pentenal	1576-87-0	C_5_H_8_O	84.1	752.6	193.924	1.11452
10	2-methylbutanal	96-17-3	C_5_H_10_O	86.1	676.9	164.694	1.15979
16	butanal	123-72-8	C_4_H_8_O	72.1	601.1	145.224	1.29144
19	2-methylpropanal	78-84-2	C_4_H_8_O	72.1	559.1	134.428	1.10159
22	(*E*,*E*)-2,4-heptadienal	4313-03-5	C_7_H_10_O	110.2	1004.2	367.235	1.61242
23	2-methyl-2-pentenal	623-36-9	C_6_H_10_O	98.1	834.6	234.949	1.16801
31	heptanal	111-71-7	C_7_H_14_O	114.2	896.2	270.49	1.67788
34	(*E*)-2-hexenal	6728-26-3	C_6_H_10_O	98.1	827.4	230.945	1.52248
38	hexanal	66-25-1	C_6_H_12_O	100.2	789.6	209.917	1.55987
41	pentanal	110-62-3	C_5_H_10_O	86.1	732.1	185.541	1.42109
44	3-methylbutanal	590-86-3	C_5_H_10_O	86.1	641.6	155.621	1.20086
49	2-methylpentanal	123-15-9	C_6_H_12_O	100.2	742.6	189.841	1.52146
55	heptanal	111-71-7	C_7_H_14_O	114.2	918.6	289.668	1.67752
59	benzaldehyde	100-52-7	C_7_H_6_O	106.1	956.4	322.013	1.14966
60	benzaldehyde	100-52-7	C_7_H_6_O	106.1	956.3	321.943	1.46584
61	2-methylpentanal	123-15-9	C_6_H_12_O	100.2	755.8	195.242	1.53061
85	3-methylbutanal	590-86-3	C_5_H_10_O	86.1	690.4	168.43	1.41855
100	(*E*)-2-hexenal	6728-26-3	C_6_H_10_O	98.1	853.1	245.282	1.16731
Alcohols							
5	2.3-butanediol	513-85-9	C_4_H_10_O_2_	90.1	814.8	223.945	1.36602
6	1.3-butanediol	107-88-0	C_4_H_10_O_2_	90.1	786.1	207.973	1.36699
14	2-methylpropanol	78-83-1	C_4_H_10_O	74.1	620.1	150.078	1.17084
15	butanol	71-36-3	C_4_H_10_O	74.1	651.3	158.113	1.39114
24	linalool	78-70-6	C_10_H_18_O	154.3	1087.9	486.194	1.21752
30	1-heptanol	111-70-6	C_7_H_16_O	116.2	971.6	335.043	1.77368
32	*n*-hexanol	111-27-3	C_6_H_14_O	102.2	885.5	263.272	1.62802
37	2-hexanol	626-93-7	C_6_H_14_O	102.2	804.3	218.077	1.574
40	2-methylbutanol	137-32-6	C_5_H_12_O	88.1	726.8	183.344	1.48425
45	2-propanethiol	75-33-2	C_3_H_8_S	76.2	608.2	147.042	1.44187
52	*tert*-butanol	75-65-0	C_4_H_10_O	74.1	538.3	129.083	1.15468
53	1-octen-3-ol	3391-86-4	C_8_H_16_O	128.2	980.4	342.589	1.15779
54	citronellol	106-22-9	C_10_H_20_O	156.3	1236.7	697.768	1.35197
56	1-octen-3-ol	3391-86-4	C_8_H_16_O	128.2	949.6	316.19	1.58839
65	2-butanol	78-92-2	C_4_H_10_O	74.1	617.8	149.49	1.3222
72	*n*-hexanol	111-27-3	C_6_H_14_O	102.2	867.3	253.174	1.63915
Esters							
2	dihydro-5-methyl-2(3H)-furanone	108-29-2	C_5_H_8_O_2_	100.1	947.8	314.6	1.41535
4	isoamyl acetate	123-92-2	C_7_H_14_O_2_	130.2	846.4	241.544	1.30701
9	propyl acetate	109-60-4	C_5_H_10_O_2_	102.1	701.7	173.04	1.16156
21	ethyl 2-methylpropanoate	97-62-1	C_6_H_12_O_2_	116.2	714.7	178.404	1.56305
28	ethyl hexanoate	123-66-0	C_8_H_16_O_2_	144.2	1002.1	364.305	1.79254
29	ethyl hexanoate	123-66-0	C_8_H_16_O_2_	144.2	1002.2	364.382	2.11443
35	ethyl trans-2-butenoate	623-70-1	C_6_H_10_O_2_	114.1	829.5	232.096	1.56569
36	ethyl 2-methylbutyrate	7452-79-1	C_7_H_14_O_2_	130.2	839.6	237.745	1.65046
39	ethyl 2-methylpropanoate	97-62-1	C_6_H_12_O_2_	116.2	744.9	190.772	1.56902
42	ethyl propanoate	105-37-3	C_5_H_10_O_2_	102.1	702	173.197	1.45433
46	ethyl acetate	141-78-6	C_4_H_8_O_2_	88.1	606.6	146.624	1.33882
47	methyl acetate	79-20-9	C_3_H_6_O_2_	74.1	523.7	125.345	1.18482
51	butyl formate	592-84-7	C_5_H_10_O_2_	102.1	724.6	182.443	1.20588
62	5-ethyldihydro-2(3H)-furanone	695-06-7	C_6_H_10_O_2_	114.1	1050.3	432.868	1.19034
67	5-ethyldihydro-2(3H)-furanone	695-06-7	C_6_H_10_O_2_	114.1	1049.6	431.743	1.5269
74	methyl heptanoate	106-73-0	C_8_H_16_O_2_	144.2	1034.8	410.735	1.35963
87	2-methyl propyl acetate	110-19-0	C_6_H_12_O_2_	116.2	748.1	192.101	1.62005
96	methyl hexanoate	106-70-7	C_7_H_14_O_2_	130.2	921.6	292.227	1.28646
Ketones							
11	2,3-pentandione	600-14-6	C_5_H_8_O_2_	100.1	691.4	168.837	1.2234
17	2-butanone	78-93-3	C_4_H_8_O	72.1	587.8	141.793	1.24607
18	2,3-butanedione	431-03-8	C_4_H_6_O_2_	86.1	574.7	138.445	1.17801
20	acetone	67-64-1	C_3_H_6_O	58.1	503.5	120.15	1.11112
26	carvone	99-49-0	C_10_H_14_O	150.2	1205.8	653.865	1.81071
43	2-pentanone	107-87-9	C_5_H_10_O	86.1	681.5	165.873	1.37289
64	5-methyl-3-heptanone	541-85-5	C_8_H_16_O	128.2	955.9	321.569	1.27667
69	3,5-dimethyl-1,2-cyclopentanedione	13494-07-0	C_7_H_10_O_2_	126.2	1050	432.323	1.18816
79	2-methyltetrahydrofuran-3-one	3188-00-9	C_5_H_8_O_2_	100.1	827.2	230.83	1.42196
83	2,3-pentandione	600-14-6	C_5_H_8_O_2_	100.1	717.8	179.646	1.28539
94	2-heptanone	110-43-0	C_7_H_14_O	114.2	888.2	264.771	1.26102
97	hydroxyacetone	116-09-6	C_3_H_6_O_2_	74.1	653.7	158.723	1.22727
99	hexan-2-one	591-78-6	C_6_H_12_O	100.2	788.9	209.539	1.17719
Acids							
12	propanoic acid	79-09-4	C_3_H_6_O_2_	74.1	699.3	172.065	1.27404
25	butanoic acid	107-92-6	C_4_H_8_O_2_	88.1	770.6	201.313	1.17355
71	hexanoic acid	142-62-1	C_6_H_12_O_2_	116.2	1034	409.581	1.62714
76	2-methylpropionic acid	79-31-2	C_4_H_8_O_2_	88.1	753	194.087	1.3722
80	butanoic acid	107-92-6	C_4_H_8_O_2_	88.1	808.5	220.425	1.16493
81	3-methylbutyric acid	503-74-2	C_5_H_10_O_2_	102.1	838.8	237.297	1.47743
93	pentanoic acid	109-52-4	C_5_H_10_O_2_	102.1	877.6	258.889	1.23545
Heterocyclics							
1	2-ethyl-3-methylpyrazine	15707-23-0	C_7_H_10_N_2_	122.2	1006.3	370.205	1.16578
13	pyrrolidine	123-75-1	C_4_H_9_N	71.1	676.9	164.694	1.27699
50	2,3,5-trimethylpyrazine	14667-55-1	C_7_H_10_N_2_	122.2	987.5	348.598	1.17267
77	methylpyrazine	109-08-0	C_5_H_6_N_2_	94.1	848.3	242.58	1.40595
78	methylpyrazine	109-08-0	C_5_H_6_N_2_	94.1	837.9	236.766	1.42044
98	2-*n*-butylfuran	4466-24-4	C_8_H_12_O	124.2	896.6	270.821	1.19839
Others							
8	acetoin	513-86-0	C_4_H_8_O_2_	88.1	738.9	188.305	1.31282
27	α-phellandrene	99-83-2	C_10_H_16_	136.2	1002.4	364.767	1.69006
48	1-octene	111-66-0	C_8_H_16_	112.2	789.5	209.831	1.46849
57	camphene	79-92-5	C_10_H_16_	136.2	950.1	316.636	1.74609
63	car-3-ene	13466-78-9	C_10_H_16_	136.2	1007.4	371.827	1.7428
68	1,8-cineole	470-82-6	C_10_H_18_O	154.3	1008.0	372.643	1.2958
70	β-ocimene	13877-91-3	C_10_H_16_	136.2	1033.8	409.291	1.2462
82	acetoin	513-86-0	C_4_H_8_O_2_	88.1	731.0	185.081	1.34384
92	dipropyl sulfide	111-47-7	C_6_H_14_S	118.2	878.6	259.428	1.15844
Unknowns							
33	unidentified	*	*	0	827	230.736	1.45433
66	unidentified	*	*	0	812.2	222.488	1.47207
73	unidentified	*	*	0	808.3	220.307	1.68882
84	unidentified	*	*	0	806.8	219.458	1.75821
86	unidentified	*	*	0	716.9	179.276	1.37992
88	unidentified	*	*	0	616.1	149.062	1.51603

^a^ Molecular weight. ^b^ Retention index. ^c^ Retention time. ^d^ Drift time.

**Table 2:** Goodness of fit (*R*^2^ (cum)) and predictive ability (*Q*^2^ (cum)) of the OPLS-DA models.

Data	Signal	LV.	*R*^2^*X* (cum)	*R*^2^*Y* (cum)	*Q*^2^ (cum)	Accuracy (%)
NR+AD Adu.	94	3	0.851	0.976	0.963	100
	51	3	0.973	0.957	0.939	100
NR+OD Adu.	94	3	0.692	0.987	0.971	100
	9	3	0.817	0.595	0.459	95
NR+SD Adu.	94	3	0.974	0.933	0.908	100
	27	3	0.897	0.988	0.976	100

## Data Availability

The original contributions presented in the study are included in the article/Supplementary Material, further inquiries can be directed to the corresponding author.

## Supplementary Materials

The following supporting information can be downloaded at: https://www.mdpi.com/article/10.3390/foods14101710/s1, Figure S1. The permutation test plots of OPLS-DA models; Figure S2. The VIP values of volatile organic compounds of OPLS-DA models.

## References

[1] Kumar R., Raj T., Nss G., Srensen M., Dhawan V. (2024). Opportunities and challenges in single-cell protein production using lignocellulosic material. Biofuels Bioprod. Biorefining.

[2] Jones S.W., Karpol A., Friedman S., Maru B.T., Tracy B.P. (2020). Recent advances in single cell protein use as a feed ingredient in aquaculture. Curr. Opin. Biotechnol..

[3] Yan J., Guo X., Li Q., Yuan X., Zhang Z., Tremblay L.A., Li Z. (2024). Biochar derivation at low temperature: A novel strategy for harmful resource usage of antibiotic mycelial dreg. Environ. Res..

[4] Chen G., Zhou T., Song Y., Yan B., Mu L., Tao J., Pei L. (2025). Alkaline hydrothermal treatment of gentamycin mycelial residues: Characteristics of disintegration, solid-state fermentation, and antibiotic resistance genes reduction. Biomass Convers. Biorefinery.

[5] Liu S., Hou X., Xin Q., Wang Y., Xin Y., Liu G., Zhou C., Liu H., Yan Q. (2022). Degradation of rifamycin from mycelial dreg by activated persulfate: Degradation efficiency and reaction kinetics. Sci. Total Environ..

[6] Melekhin A.O., Tolmacheva V.V., Goncharov N.O., Apyari V.V., Dmitrienko S.G., Shubina E.G., Grudev A.I. (2022). Multi-class, multi-residue determination of 132 veterinary drugs in milk by magnetic solid-phase extraction based on magnetic hypercrosslinked polystyrene prior to their determination by high-performance liquid chromatography-tandem mass spectrometry. Food Chem..

[7] Wang B., Wang Y., Xie X., Diao Z., Xie K., Zhang G., Zhang T., Dai G. (2020). Quantitative analysis of spectinomycin and lincomycin in poultry eggs by accelerated solvent extraction coupled with gas chromatography tandem mass spectrometry. Foods.

[8] Mehrabi A., Mahmoudi R., Morasa H.K., Norian R., Mosavi S., Ahmadi Z., Kazemi M., Alizadeh A. (2022). Evaluation of sulfonamide antibiotic residues of honey samples produced in different regions of qazvin province by ELISA. J. Chem. Health Risks.

[9] Li S., Fan X., Wu Y., Liao K., Huang Y., Han L., Liu X., Yang Z. (2021). A novel analytical strategy for discriminating antibiotic mycelial residue adulteration in feed based on atr-ir and microscopic infrared imaging. Spectrochim. Acta Part A Mol. Biomol. Spectrosc..

[10] Ge C., Yang Z., Fan X., Huang Y., Shi Z., Zhang X., Han L. (2024). A new spectral simulating method based on near-infrared hyperspectral imaging for evaluation of antibiotic mycelia residues in protein feeds. Spectrochim. Acta Part A Mol. Biomol. Spectrosc..

[11] Rodríguez S.D., Rolandelli G., Buera M.P. (2019). Detection of quinoa flour adulteration by means of FT-MIR spectroscopy combined with chemometric methods. Food Chem..

[12] Yang X., Lorjaroenphon Y., Li H., Cadwallader K.R., Wang X., Zhang Y. (2018). Quantification of odorants in animal feeds at commercial swine and poultry operations. Trans. ASABE.

[13] Gu S., Zhang J., Wang J., Wang X., Du D. (2021). Recent development of HS-GC-IMS technology in rapid and non-destructive detection of quality and contamination in agri-food products. TrAC Trends Anal. Chem..

[14] Kranenburg R.F., Verduin J., Stuyver L.I., Ridder R.D., Beek A.V., Colmsee E., Asten A.V. (2020). Benefits of derivatization in GC-MS-based identification of new psychoactive substances. Forensic Chem..

[15] Hernández-Mesa M., Ropartz D., García-Campaña A.M., Rogniaux H., Dervilly-Pinel G., Le Bizec B. (2019). Ion Mobility Spectrometry in Food Analysis: Principles, Current Applications and Future Trends. Molecules.

[16] Yang X., Zhang T., Yang D., Xie J. (2023). Application of gas chromatography-ion mobility spectrometry in the analysis of food volatile components. Acta Chromatogr..

[17] Tian H., Xiong J., Chen S., Yu H., Chen C., Huang J., Yuan H., Lou X. (2023). Rapid identification of adulteration in raw bovine milk with soymilk by electronic nose and headspace-gas chromatography ion-mobility spectrometry. Food Chem. X.

[18] Arroyo-Manzanares N., García-Nicolás M., Castell A., Campillo N., Viñas P., López-García I., Hernández-Córdoba M. (2019). Untargeted headspace gas chromatography—Ion mobility spectrometry analysis for detection of adulterated honey. Talanta.

[19] Dou X., Zhang L., Yang R., Wang X., Yu L., Yue X., Ma F., Mao J., Wang X., Li P. (2022). Adulteration detection of essence in sesame oil based on headspace gas chromatography-ion mobility spectrometry. Food Chem..

[20] Calle J.L.P., Vázquez-Espinosa M., Barea-Sepúlveda M., Ruiz-Rodríguez A., Ferreiro-González M., Palma M. (2023). Novel Method Based on Ion Mobility Spectrometry Combined with Machine Learning for the Discrimination of Fruit Juices. Foods.

[21] Yang X., Xing B., Guo Y., Wang S., Guo H., Qin P., Hou C., Ren G. (2022). Rapid, accurate and simply-operated determination of laboratory-made adulteration of quinoa flour with rice flour and wheat flour by headspace gas chromatography-ion mobility spectrometry. LWT—Food Sci. Technol..

[22] Song Y., Guo T., Liu S., Gao Y., Wang Y. (2022). Identification of Polygonati Rhizoma in three species and from different producing areas of each species using HS-GC-IMS. LWT—Food Sci. Technol..

[23] Yang X., Zhu K., Guo H., Geng Y., Lv W., Wang S., Guo Y., Qin P., Ren G. (2021). Characterization of volatile compounds in differently coloured Chenopodium quinoa seeds before and after cooking by headspace-gas chromatography-ion mobility spectrometry. Food Chem..

[24] Zhang P.P., Gui X.J., Fan X.H., Li H., Li H.Y., Li X.P., Dong F.Y., Wang Y.L., Yao J., Shi J.H. (2025). Quality identification of *Amomi fructus* using E-nose, HS-GC-IMS, and intelligent data fusion methods. Front. Chem..

[25] Trygg J., Wold S. (2002). Orthogonal projections to latent structures (O-PLS). J. Chemom..

[26] Bylesj M., Rantalainen M., Cloarec O., Nicholson J.K., Holmes E., Tryg J. (2006). OPLS discriminant analysis: Combining the strengths of PLS-DA and SIMCA classification. J. Chemom..

[27] Rocamora-Rivera B., Arroyo-Manzanares N., Viñas P. (2024). Detection of Adulterated Oregano Samples Using Untargeted Headspace–Gas Chromatography–Ion Mobility Spectrometry Analysis. Foods.

[28] Xiong Y., Zheng X., Tian X., Wang C., Chen J., Zhou L., Xu D., Wang J., Gilard V., Wu M. (2024). Comparative study of volatile organic compound profiles in aromatic and non-aromatic rice cultivars using HS-GC-IMS and their correlation with sensory evaluation. LWT—Food Sci. Technol..

[29] Wen Y., Li Z., Ning Y., Yan Y., Li Z., Wang N., Wang H. (2024). Portable Raman spectroscopy coupled with PLSR analysis for monitoring and predicting of the quality of fresh-cut Chinese yam at different storage temperatures. Spectrochim. Acta Part A Mol. Biomol. Spectrosc..

