# Supplementary material for: Rapid Detection of Antibiotic Mycelial Dregs Adulteration in Single-Cell Protein Feed by HS-GC-IMS and Chemometrics
Source: Foods. 2025 May 12;14(10):1710. doi: 10.3390/foods14101710 (PMC12111289; doi:10.3390/foods14101710)
Supplement: Supplementary file 1 [file foods-14-01710-s001.zip › foods-3615003-supplementary.pdf]

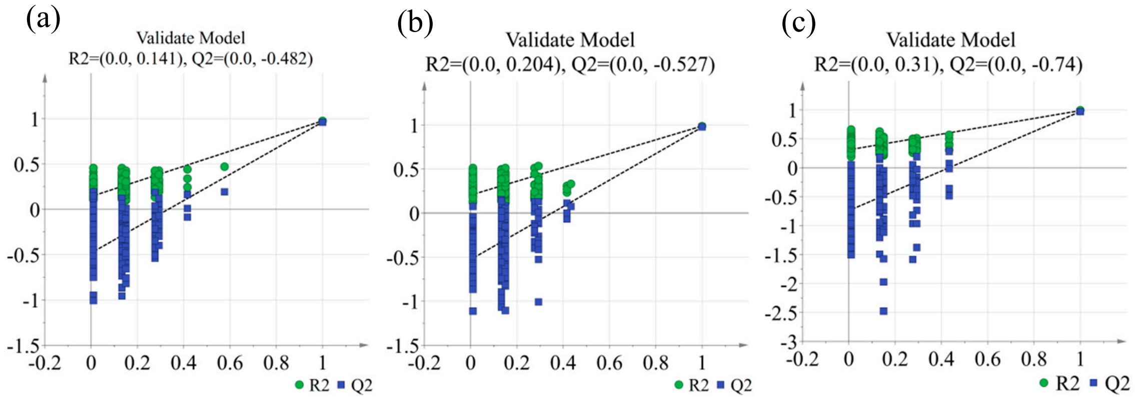

Figure S1. The permutation test plots of OPLS-DA models. (a) AD-adulterated samples, (b) SD-adulterated samples, and (c) OD-adulterated samples.

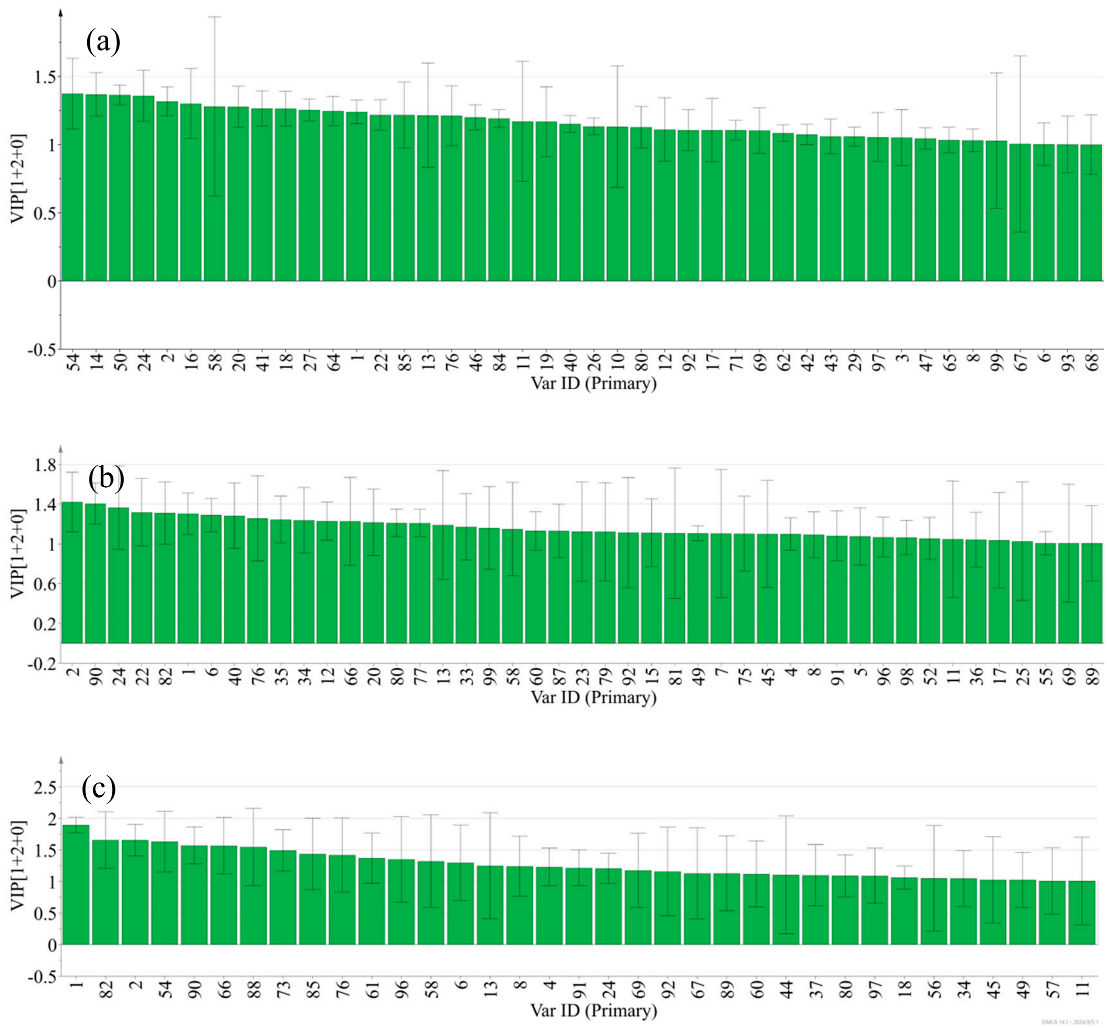

Figure S2. The VIP values of volatile organic compounds of OPLS-DA models. (a) AD-adulterated samples, (b) SD-adulterated samples, and (c) OD-adulterated samples.
